# The use and impact of quality of life assessment tools in clinical care settings for cancer patients, with a particular emphasis on brain cancer: insights from a systematic review and stakeholder consultations

**DOI:** 10.1007/s11136-016-1278-6

**Published:** 2016-04-02

**Authors:** Sarah King, Josephine Exley, Sarah Parks, Sarah Ball, Teresa Bienkowska-Gibbs, Calum MacLure, Emma Harte, Katherine Stewart, Jody Larkin, Andrew Bottomley, Sonja Marjanovic

**Affiliations:** 1 RAND Europe, Westbrook Centre, Cambridge, CB4 1YG UK; 2 RAND Corporation, Pittsburgh, PA USA; 3 European Organisation for Research and Treatment of Cancer (EORTC), Brussels, Belgium

**Keywords:** Quality of life, Oncology, Brain cancer

## Abstract

**Purpose:**

Patient-reported data are playing an increasing role in health care. In oncology, data from quality of life (QoL) assessment tools may be particularly important for those with limited survival prospects, where treatments aim to prolong survival while maintaining or improving QoL. This paper examines the use and impact of using QoL measures on health care of cancer patients within a clinical setting, particularly those with brain cancer. It also examines facilitators and challenges, and provides implications for policy and practice.

**Design:**

We conducted a systematic literature review, 15 expert interviews and a consultation at an international summit.

**Results:**

The systematic review found no relevant intervention studies specifically in brain cancer patients, and after expanding our search to include other cancers, 15 relevant studies were identified. The evidence on the effectiveness of using QoL tools was inconsistent for patient management, but somewhat more consistent in favour of improving patient–physician communication. Interviews identified unharnessed potential and growing interest in QoL tool use and associated challenges to address.

**Conclusion:**

Our findings suggest that the use of QoL tools in cancer patients may improve patient–physician communication and have the potential to improve care, but the tools are not currently widely used in clinical practice (in brain cancer nor some other cancer contexts) although they are in clinical trials. There is a need for further research and stakeholder engagement on how QoL tools can achieve most impact across cancer and patient contexts. There is also a need for policy, health professional, research and patient communities to strengthen information exchange and debate, support awareness raising and provide training on tool design, use and interpretation.

**Electronic supplementary material:**

The online version of this article (doi:10.1007/s11136-016-1278-6) contains supplementary material, which is available to authorized users.

## Introduction

In many brain cancer patients, current treatment options are not curative, focussing instead on prolonging survival while maintaining or improving patients’ quality of life (QoL) [1, 2]. There is often a need to balance the benefits of extended survival or delayed progression with the potential negative effects of treatment on QoL [3]. As new and more targeted treatments are developed, with increased risk of severe side effects and neurotoxicity, the importance of considering QoL as an outcome increases even further [4].

QoL, in this patient group, is measured using a wide range of instruments/tools [1, 2, 5]. Tools available range from those which measure generic quality of life aspects (e.g. the SF 36 and the Nottingham Health Profile) to cancer-specific tools and brain cancer-/tumour-specific questionnaires within these. Some tools focus on many aspects of QoL, while others focus on specific functions or symptoms. For example, the Karnofsky performance status scale focuses on functional performance while EORTC and FACT-br tools cover various physical, role, emotional, cognitive and social functioning and diverse symptoms. Tools are often modular, incorporating a general (core) questionnaire, for use with all cancer patient groups, supplemented by a brain cancer-/tumour-specific questionnaire (module), which focuses on issues of particular relevance to this patient group. For an overview of tools, see Table S1 in the supplementary files.

The use of these tools in the measurement of QoL as an outcome in clinical trials has become increasingly important, in addition to survival [1, 2, 5–11]. As well as using QoL in clinical trials, a number of authors have also hypothesised that QOL data could support more inclusive clinical decision-making on care regimes and management, by providing patients’ perspectives on their care [5, 12]. However, there is a lack of knowledge on how the use of QoL instruments could lead to changes in clinical decision-making and patient management [5, 13, 14].

We undertook a systematic review to examine the impact of using QoL tools on the health care of brain cancer patients in clinical settings. In addition, we undertook key informant interviews and stakeholder consultations in order to explore why tools are, or are not, used in care contexts, and enablers and barriers to their use. Based on this evidence, we suggest potential implications for future policy and oncology practice.

## Design

### Systematic review

The systematic review was conducted according to published guidance for systematic reviews of health interventions [15] and is reported in line with PRISMA (Preferred Reporting Items for Systematic Reviews and Meta-analyses) guidelines [16].

Any type of study that evaluated the use of a QoL tool as an intervention in routine clinical practice, and/or used QoL data in order to make a change in patient care, was eligible for inclusion. Initially, participants of interest were restricted to patients with brain cancer. However, we found no relevant studies exclusively in this patient group. Given the paucity of this literature, we expanded the search to consider all relevant interventions within all oncology settings, except a surgical oncological setting,^1^ with a view to identifying potentially transferable insights to inform a future research agenda.

Primary outcomes of interest related to change in care management. They included, but were not limited to, patient–physician communication, patient treatment adherence or clinical decision-making/clinical management/changes in treatment pathways. Studies that only examined the feasibility, validity, reliability or acceptability of QoL tools were not included. Studies that examined QoL for predictive purposes, such as survival,^2^ were also not included. The search strategy, study selection, data extraction and risk of bias assessment methods are presented in Table 1.

**Table 1:** Systematic review search strategy, study selection, data extraction and risk of bias assessment

Search strategy	Electronic searches of four research literature databases (PubMed, EMBASE, Cochrane (SR & Trials), Web of Science (SCI)) were conducted from 2000 up to 15 July 2015. In addition, searches for grey literature were conducted in OAIster, OpenGrey, NYAM Grey Literature Report and Lexis Nexis, up to 11 September 2015. Details of the electronic searches are provided in supplementary resource S2. Searches of eligible studies’ reference lists and forward citation tracking (using Google Scholar and PubMed) were also conducted. Initially, no language or date restrictions were applied, but due the large number of references identified, the search was restricted to English language publications and to full publications (i.e. not conference abstracts)
Study selection and data extraction	Retrieved title–abstract records were initially screened by one of several reviewers (SK, JE, SB, TB-G or EH) against the PICOS criteria. A random sample of 10 % of records was independently screened by a second reviewer. Full-text screening of potentially eligible study reports was undertaken by at least two reviewers working independently, with any discrepancies resolved by a third reviewer. Study data were extracted by one reviewer and checked by a second reviewer
Risk of bias assessment	Risk of bias assessments were done by one reviewer and checked by a second reviewer. For the RCTs, the Cochrane Risk of Bias tool [15] was used to assess potential bias in studies for each outcome; six criteria were considered: random sequence generation, allocation concealment, blinding of participants, personnel (where feasible) and outcome assessors, baseline comparability between groups, incomplete outcome data, and selective outcome reporting. Risk of bias judgements in domains judged most critical (sequence generation, allocation concealment, incomplete outcome data) was used to determine a summary study-level risk of bias. For cohort studies, and pre–post studies, we used criteria published on websites [17, 18]. Lastly, we used GRADE criteria [19] to enable assessment of the quality of the body of evidence for each outcome

Studies were summarised in a narrative synthesis. Where data allowed, we computed study-level standardised mean differences (SMD) between comparison groups with 95 % confidence intervals. Visual presentation of these results is presented in forest plots. Due to clinical heterogeneity between the studies, meta-analyses were not conducted.

### Key informant interviews

**Fig. 1:**
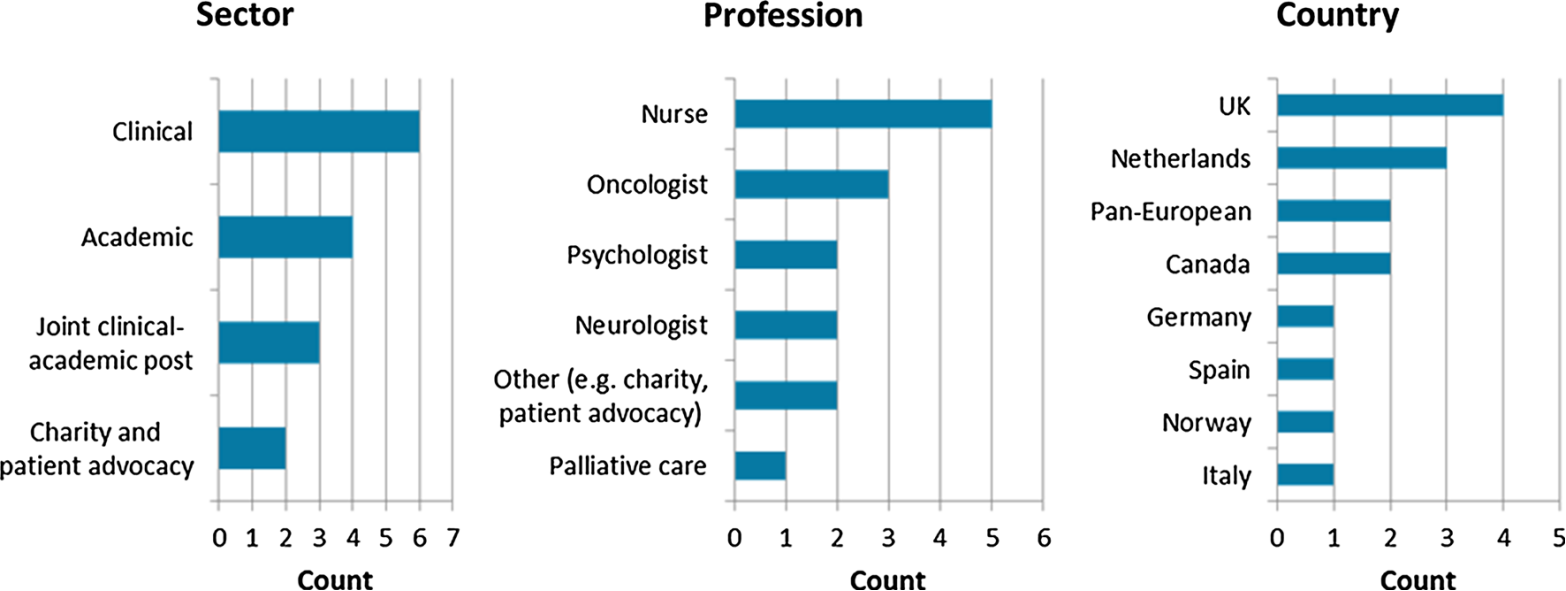
Profiles of interviewees broken down by sector, country and profession

To complement and expand on the evidence from the systematic review, we conducted telephone interviews with professionals on quality of life and/or brain cancer and advocacy group representatives from European and North American countries, as well as pan-European organisations. We interviewed individuals who had experience implementing and/or conducting research on QoL tools in patients with brain cancer, many of whom could also comment on what QoL tools are used across various types of cancers. Interviewees were identified through a combination of publication research and a snow-balling approach. They were invited by email, with an explanation of the background to the study. Interviews were conducted by a single researcher (SM, SP, EH, CML, JE) following a semi-structured format and lasted 30–60 min. With consent, interviews were audio-recorded and transcribed. Interview data were coded by a single researcher (SP) and checked by a second researcher (SM), guided by initial interview themes. Additional themes were added as they emerged from the data. The interviews were rooted in an approach combining questions exploring particular themes (but not driven by any positivist hypotheses on the themes) and also allowing for emerging issues to be captured, coded and grouped into analytical constructs—thus, the analysis followed principles rooted in grounded theory [20]. In total, we conducted 15 interviews between July and October 2015. The professional and sector background of those interviewed, as well as country background, is represented in Fig. 1 Profiles of interviewees broken down by sector, country and profession.

### Stakeholder consultation at the IBTA summit

We undertook a stakeholder consultation at the International Brain Tumour Alliance (IBTA) Second World Summit of Brain Tumour Patient Advocates (October 2015). We consulted representatives on issues explored in the interviews, during plenary and parallel sessions, and discussed our emerging findings. The majority of participants were from patient advocacy groups, patient support groups and research funding groups; academics, clinicians and industry representatives were also present. We also received written feedback from 19 representatives. Data were coded and triangulated against insights from the interview data.

## Results

### The impact of QoL assessment tools in routine practice in oncology settings

**Fig. 2:**
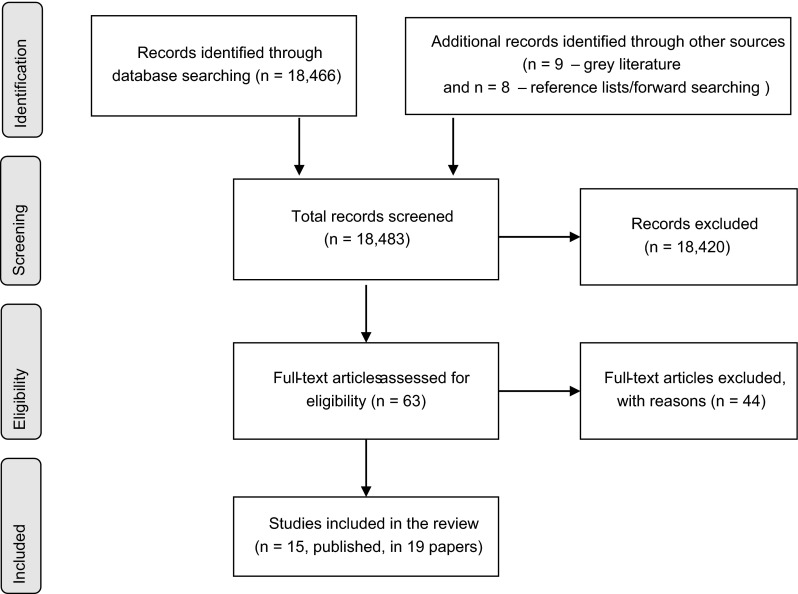
PRISMA flow diagram of the flow of citations reviewed during the systematic review

The systematic review identified 15 studies (reported in 19 articles) on the use of QoL assessment tools in clinical cancer care settings.^3^ Three of the studies were considered to have a low risk of bias [21–24], with the remaining studies having either an “unclear” or “high risk” of bias (see supplementary table S3). Figure 2 illustrates the flow of studies through the systematic review process.

Of the 13 studies conducted in adults, different cancer patient groups were represented including those with lung cancer [23, 25, 26], prostate and/or breast cancer [22, 27, 28] or various types of cancers [21, 29–34], including head cancer [29]. The two studies conducted in children and teens included various types of cancers, including brain tumours [24, 35].

Nine of the studies examined the effectiveness of the European Organisation for Research and Treatment of Cancer QoL Questionnaire C30 (EORTC QLQ-30) questionnaire. The remaining six studies variously examined the Functional Assessment of Cancer Therapy—General (FACT-G), Prostate QOL (PROSQoLI), QoL in Childhood Oncology (QLIC-ON PROfile), Pediatric QoL and Evaluation of Symptom Technology (PediQUEST), Electronic Self-report Assessment-Cancer (ESRA-C) and the Patient-Reported Outcomes Measurement Information System (PROMIS). Four types of outcomes dominated the literature, which are presented below. Details of the characteristics of the included studies and their results are presented in Table S3 (Supplementary files).

### Physician (or nurse)–patient communication

**Fig. 3:**
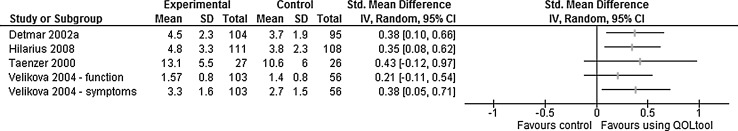
Studies that evaluated the frequency of QoL issues discussed during consultation

Ten studies^4^ assessed this outcome [21, 23, 25, 27–30, 33–35]. Four studies examined the frequency with which QoL was discussed, with consistency shown in favour of using a QoL tool to improve communication (Fig. 3). Eight studies examined more specific QoL issues; the issues assessed, and the results, were inconsistent across studies. Those found to be significantly more frequently discussed during consultation in the intervention groups compared with the control groups include social functioning, fatigue and dyspnoea [30], emotional functioning [23, 29, 33, 35], psychosocial functioning [35] and daily activities [33].

### Patient management

**Fig. 4:**
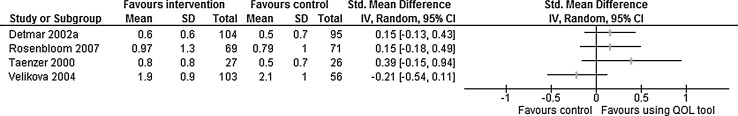
Studies that evaluated the numbers of actions/medical decisions taken during consultation

Twelve studies^5^ reported on outcomes related to patient management [21–24, 26–28, 30, 32–35]. Four reported on the number of actions/medical decisions taken during consultations, none of which found significant effects (Fig. 4). An additional study reported that the proportion of patients who received at least one therapeutic option for QoL therapy did not differ between the intervention and control groups [22].

Three studies examined the number of medical actions taken for different types of QoL domains/issues (such as emotional concerns) during consultations, but very few significant effects were observed (Table 2). Four studies reported on specific actions taken during consultations. In these studies, no significant effects were observed between intervention and control groups in the frequency of medication prescription, referrals, test ordering or modification/cessation of chemotherapy, and inconsistent effects were observed for counselling (Table 2).

**Table 2:** Number of medical actions taken and actions taken for specific domains/issues as identified in the systematic review

Outcome	Overview of results
Number of actions taken for specific domains/issues (*n* = 3 studies)	Of two studies that reported on the number of actions taken for specific domains, only Nicklasson et al. [23] reported that the number of diagnostic and therapeutic interventions directed to emotional concerns (SMD 0.45 [95 % CI 0.15–0.76]) and social concerns (SMD 0.38 [95 % CI 0.08–0.69]) were larger in the intervention group compared to the control group. In addition, a study by Wolfe et al. [24] reported that the QoL intervention led to the initiation of a psychosocial (56 %), pain (34 %), social work (33 %) or palliative care (29 %), consult, but more detailed data were not reported
Number of specific actions (*n* = 4 studies)	No studies reported differences between intervention and control groups for prescription of medications [21, 30], ordering of tests [21, 30], referrals [21, 35] or modification/cessation of chemotherapy [21]. Of three studies that evaluated advice/counselling as an outcome [21, 22, 30], only Detmar et al. [30] found that a statistically greater percentage of patients in the intervention group received counselling from their physician on how to manage their health problems compared to those in the control group (23 vs. 16 %)

**Table 3:** QoL tools with potential applicability to a brain cancer care context mentioned by interviewees

Category	QoL tool name
Brain cancer specific	EORTC QLQ-BN 20
	FACT-br
	FACT-mng
	Patient concerns inventory
	M.D. Anderson Symptom Inventory-Brain Tumor Module
Cancer specific	Distress thermometer
	Macmillan holistic needs assessment
	Distress Assessment & Response Tool (DART)
	Psychological Screen for Cancer (PSCAN)
General	EQ-5D
	SF-36
	Karnofsky Performance Status scale
	Barthel Index

**Table 4:** Factors influencing the use of QoL tools in cancer care, identified in interviews

	Facilitators	Challenges
System-level factors	A formal requirement for use in trials can influence care of patients participating in trials and patients in settings where trials take place Availability of human resources (e.g. trained nurses and doctors to administer and interpret findings) Value placed on patient perspectives and patient engagement by the health system Although rare, the presence of guidelines on tool use^a^	A general lack of policy and guidelines for the use of QoL instruments in cancer care contexts A need for training of health professionals, confounded by health system resource constraints and time demands Challenges to effectively communicating QoL findings from clinical trials to the point of prescription A lack of insights on the place of QoL assessment findings in valuations of drugs
Tool-related factors	A relatively simple design is important in a care context (with simplicity being relatively less important in trial contexts where the capacity and resources to cope with more detailed instruments is better established) Ability of questions to address aspects of QoL that are meaningful to the patient and the clinician (this are not mutually exclusive but not always the same in priority) Scope for remote administration (e.g. iPads, telephone administration) but requires reliable devices, software and training	No “gold standard”: perceptions of overly complex or overly simple designs and the associated need for some standardisation in trials but sufficient tailoring for care contexts (across patient profiles and cancer types and stages) Cultural specificities associated with HRQoL and implications on the nature of questions that need to be asked Having patients complete (and staff process) different forms for different cancers was seen to potentially be too burdensome
Administration, data and disease-related factors	Follow-up on findings with patient/carer as key for public acceptability of tools and their take-up	A general lack of awareness about the diversity of available tools and how to access and engage with them, among cancer patients
	Scope for proxy-reporting (but not without trade-offs) when patients are unable to complete questionnaires directly (e.g. due to issues such as cognitive decline)	Shifting patient expectations of HR QoL during the course of disease Patient versus proxy views can be different

^a^For example, the National Comprehensive Cancer Network (NCCN) Clinical Practice Guidelines in Oncology have a guideline on the use of the distress thermometer

In addition to these two main outcomes, secondary outcome measures reported included the impact of the use of QoL tools as part of the consultation process on patient well-being and on patient satisfaction with their care/treatment. Overall, there was evidence to suggest that the use of tools had no, or a very small, effect on either patient QoL or patient satisfaction.

In the rest of the paper, we draw on interview findings to explore the current use of QoL tools in healthcare contexts with a particular emphasis on brain cancer, enablers and barriers to their use and implications for the future. This evidence is supplemented with insights from stakeholder consultation at the IBTA summit and jointly expands on and complements the findings from the systematic review. The primarily focus was on the brain cancer context, but also—in line with an inductive approach—revealed some wider reaching insights of relevance across different types of cancers. The aim of the interviews was to scope a diverse range of issues that could inform further studies. In line with this, and especially considering the scarcity of studies on the use of QoL tools as an intervention in cancer care, we did not aim to quantify the strength of different responses in this aspect of the study, but rather to capture the diversity of issues perceived to be important and relevant.

### Current use of QoL assessment tools in healthcare delivery for cancer patients, with particular emphasis on brain cancer

Although interview evidence identified some cases of the use of QoL tools in the treatment and care of patients with brain cancer (see Box 1),^6^ their use in clinical contexts is reportedly rare, both for brain cancer and wider cancer care. Where QoL tools are used, this was cited to be on the initiative of a key individual. Overall interviewees noted that QoL tools are principally used within clinical trials, and many of the tools used in the care of brain cancer patients differ to the tools used in clinical trials (a matter we return to later in the paper). However, interviewees pointed to opportunities for “spillover effects” from the use of QoL assessment tools in the care of patients participating in trials to other patients in the same clinical setting.^7^

**Box 1:** Specific examples of how QoL assessment tools are being used in the care of cancer patients, including brain cancer as reported in interviews

Tool used in care delivery, as reported in interviews	Use
Distress Thermometer	Used in the care of patients with a variety of cancers including brain cancer, in some neuro-oncology settings in the Netherlands. Dutch guidelines for psychosocial care of cancer patients recommend the use of QoL measures, and, as one interviewee noted, although it “… is not a real QoL instrument… it makes it possible to discuss QoL with patients… and can help to detect complaints and issues”. (INT04). This in turn was seen to facilitate more appropriate referral pathways
Distress Assessment and Response Tool (DART)	Mandated by the provincial government of Ontario as a general cancer QoL measure for assessing all patients in cancer centres in Ontario, Canada. It is administered each time a patient visits the clinic (unless they are undergoing daily radiation treatment). The data are used by clinicians who scan the results before meeting patients and can ask patients specific questions that warrant attention in follow-up
Psychological Screen for Cancer (PSCAN)	Used by the British Columbia Cancer Agency in Canada, as part of its Patient and Family Counselling Services Initiative, as it covers issues they feel counselling can help with. It is currently administered to all patients at their first visit, and they are trialling using it repeatedly during a patient’s care
Barthel Index	Used in clinical practice in some settings in Spain to gather information on the status of a brain cancer patient, in terms of performance in activities of daily living

The tools listed are examples provided by interviewees. There was reported to be a debate as to what is considered an official QoL assessment tool. As such, the tools listed might not be recognised as a formal QoL assessment tool by some professions, and their use may be limited to specific settings

Despite the reported low levels of routine use at present, there was broad consensus among interviewees that QoL tools have unharnessed potential to “*improve daily care*”.^8^ This was perceived to be exemplified by the fact that both the International Society for Quality of Life Research and the International Society for Pharmacoeconomics and Outcomes Research are investigating the use in the clinical practice setting.^9^ The principal benefits reported by interviewees related to diverse aspects of improved patient–physician communication in both brain cancer and wider cancer care contexts. For example, interviewees considered that QoL outcomes provide clinicians with a better understanding of patients’ perspective,^10^,^11^ encouraging discussions that enable clinicians to take a more holistic view of patients’ needs.^12^ For patient advocacy group representatives consulted, their particularly interest in QoL tools related to their potential to help healthcare professionals understand patient needs, across cancer contexts.

“QoL is an important outcome as it tells you how the patient feels and we know there is a difference between clinical parameters and subjective patient parameters. Someone can be terribly sick but may not feel it that way and vice versa. QoL is about the patient perspective and the functioning of the patient in his or her entirety”.^13^

Interviewees also suggested that QoL tools could help empower patients to play a more informed role in decision-making through a greater understanding of the implications of particular treatment on their QoL. This was considered to be particularly pertinent to brain cancer care, given that treatment is not curative.^14^, ^15^

“There is a particular concern with QoL in the brain tumour field because there are no cures for many of the diseases covered. Where there is no cure, the priority is to minimise harm done to the patient by treatments”.^16^

In addition to patient–physician communication,^17^ interviewees identified additional potential uses including using the tools to help assess the effects of environmental changes in the clinic,^18^ or to help inform the most appropriate ways of delivering and communicating results of scans^19^ to patients. During stakeholder consultation wider uses of QoL were noted, including their use as an advocacy tool in discussions about valuing brain cancer treatments with regulators, which highlights the need for more effective communication of findings from clinical trials to decision-makers.^20^ For example, it was highlighted that QoL results from trials were often not at the forefront of trial findings, suggesting scope for more user-friendly means of communication.

### Factors influencing the use of QoL assessment tools in cancer care, with a particular emphasis on brain cancer

A range of facilitators and challenges associated with the use of QoL tools were discussed by interviewees. These span system-level, tool-, administration- and data-related factors (Table 4). While some factors apply across all cancers, others are disease or patient profile specific. For example, a particular issue affecting the measurement of QoL in brain cancer patients is the impact of neurocognitive decline on the patients’ ability to complete questionnaires. Instead QoL questionnaires may have to be completed or supported by a proxy. This was considered to have implications for the value of the data produced.^21^ A proxy may have a different interpretation of the patient’s QoL, or a patient may respond differently compared to when alone.^22^ Both situations could lead to bias.^23^ Another feature of neurocognitive decline is that patients may not be aware they are experiencing it and therefore may not be able to report it on a QoL questionnaire.^24^

Other drivers of use are more universal. There was perceived to still be a low level of awareness of the utility of QoL tools among clinicians in oncology settings. Although this awareness has somewhat increased, in part facilitated by the inclusion of QoL sessions at conferences and greater acceptance of QoL-related research in academic journals,^25^ there is scope for more awareness raising. A number of interviewees pointed out that although clinical trial protocols generally require that the impact of a treatment on patients’ QoL be reported alongside clinical outcomes, it is predominantly still considered a secondary outcome.^26^^,^^27^ As such, interviewees reflected that QoL data are sometimes published in lower impact journals and at a later point in time, which reduces its visibility.

Interviewees also raised issues related to the administration and interpretation of QoL data. This included a lack of time to administer the measures, as well as time to interpret the results and discuss with patients. Two interviewees suggested that having individuals devoted to administering QoL could help to overcome this challenge, but that it also requires training for clinicians to be able to understand QoL data, which is generally presented as a series of numerical scores, and to be able to interpret what this means for the particular patient. Current training was considered to be insufficient.

“Training is particularly important, as it is difficult to interpret patients’ responses – clinicians are used to looking at physical symptoms and find it challenging to adapt to incorporating patients’ assessment of their symptoms into their decision-making”.^28^

The subjective nature of QoL assessment also affects ease of interpretation, as different patients will differ in their expectations of QoL and expectations may also alter as the disease progresses, so tools may not remain equally applicable across disease stages.^29^

The number of tools available also poses both opportunities for patient-centred and bespoke instruments, but also some challenges. It was apparent from interviews that there is no “gold standard” and interviewees discussed a range of tools used (Table 3), with notable differences in opinions between interviewees as to what constitutes an official QoL assessment tool and what are more informal instruments.^30^ In general, there was consensus across interviews that in brain cancer care contexts general QoL tools are used more often than cancer-specific tools, with latter being used more frequently in clinical trials. In clinical trials, it was perceived that the infrastructure and resource capacity to administer and interpret more detailed cancer-specific tools are better established which may help explain the greater recourse to cancer-specific QoL questionnaires in trial contexts. One interviewee suggested that general tools are more practical in oncology care settings as they can be used across cancers, and more familiar to healthcare staff, a view supported during the stakeholder consultation. Two interviewees, however, raised concerns about the utility of non-disease specific measures in providing insights on the distinct needs and concerns of a particular patient segment.^31^

## Discussion and conclusions

In our systematic review, we examined the impact of QoL assessment tools in clinical cancer care settings, but did not find any studies exclusively in brain cancer patients. Based on published studies that included various other types of cancer patients, there is some evidence to suggest that QoL data may improve patient–physician communication, and that emotional functioning in particular *may* be discussed more frequently during consultation after implementation of a QoL tool. Our finding is consistent with the wider literature [1, 5, 10], which suggests that QoL tools enable both doctors and patients to discuss more sensitive issues and/or focus discussions on non-medical issues identified as important by the patient [29, 36]. It is likely that these findings are applicable to brain cancer patients, although this merits further research.

It is not clear, however, whether the systematic review evidence regarding the number of actions/medical decisions, or QoL as an outcome, is applicable to brain cancer patients specifically. Whereas the systematic review identified insights which may be transferable to brain cancer contexts, this calls for further research, and particularly given the notable gaps in the current literature on brain cancer on this topic.

However, insights from our interviews and wider consultation identified a diverse range of factors influencing the use of QoL instruments in brain cancer care and cancer care more widely, as overviewed in Table 4. As such, these insights contribute to addressing the scarcity of evidence in the literature. They also point to key areas for policy consideration to do with communication and information exchange, capacity building and regulation.

There was widespread belief among consulted experts that QoL assessment tools have a beneficial role to play in improving clinical practice through more inclusive decision-making, despite a range of challenges that are yet to be addressed. Many of these were seen to apply across cancer contexts, but some were seen to be more brain cancer specific. For example, given that some brain cancer patients’ treatment is not curative, interviewees suggested that brain cancer patients and their physicians might place more importance on QoL when making decisions, than those patients who have better survival prospects. Findings from the systematic review might therefore underestimate the impact of QoL on medical actions taken in this particular group of patients.

Factors related to time constraints to the use of tools^32^ and a need for establishing guidelines on tool use and interpretation^33^ were seen to apply more widely across cancer contexts and shed light on some capacity-building priorities for future practice. Interviewees also suggested that clinicians struggle to interpret QoL data and that there are risks with QoL tools identifying issues beyond the physicians’ perceived duty of care and/or control—as a result, physicians may feel powerless to act on the data [28, 29, 37]. This suggests that there is a need for training on efficient and effective tool use, and for information exchange between healthcare professionals [28, 34, 38, 39], patient groups and wider stakeholders, on experiences of good practice. This includes continuing the debate on the benefits and limitations of different tools, how to use them and adapt them, how to interpret the data and how to empower patients’ to engage with QoL tools and discussions so that they can make the most informed treatment decision based on their own needs.

Closely related to these issues are challenges to managing a requisite degree of tool standardisation for clinical trial purposes, with scope for customisation in care to ensure patient-centred instruments [13, 14, 40]. A future research agenda might explore further evaluations of modular tools tailored to specific diseases, cultural contexts and patient profiles (e.g. paediatrics, end-of-life care). There is also a need to better understand how QoL assessment tools can be used effectively across multidisciplinary healthcare teams.

Finally, a need for more effective, user-friendly and consistent ways of communicating QoL findings from clinical trials to regulators and to clinicians was also highlighted in our consultations as an area for consideration in oncology policy. This was partly seen to relate to the fact that QoL outcomes are considered secondary outcome measures in clinical trials, often driven by funding body requirements rather than an academic or clinician interest.^34^ Such issues have been discussed in contexts outside of cancer care as well [41]. In this light and in view of increasing government policy focus on patient involvement in care [13], there is a need for more discussion with regulators around the types of policies and guidelines for the administration and use of QoL tools [14], the place of QoL in value based assessments and on the health economics of QoL, and how to most effectively communicate QoL assessment results from clinical trials to the those making prescription decisions, both regulators and clinicians. In addition, the literature suggests that QoL measures might be most useful for care where they come with specific recommendations for changing care or decision guidelines to help clinicians translate the scores [5, 32].

There are some limitations to consider regarding the evidence included in the systematic review, and in the review itself. First despite the number of RCTs identified in the systematic review, the majority of included studies were at high risk of bias due to a lack of blinding and/or high drop-outs rates with “as-treated” analysis done. In addition, a number of studies had small sample sizes and were conducted among high functioning individuals. Given these methodological limitations, the reliability of some of the results is uncertain. In addition, there were methodological differences between studies in the definition and scope of QoL domains measured, assessment tools used, clinical settings and outcomes reported, making comparison of results between studies difficult. These methodological limitations have been widely acknowledged elsewhere [1, 5, 42, 43], suggesting that trials need to be further standardised to allow a more robust analysis of the use of QoL tools in clinical practice. However, the need for standardisation in trials needs to weighed against a degree of customisation in care to ensure patient-centred instruments that facilitate patient-centred care [13, 14, 40].

Regarding the systematic review, we took a number of steps to minimise selection and reviewer biases, but it is possible that some relevant studies may have been missed, such as those published in non-English languages. Moreover, our review inclusion/exclusion criteria were very focused, and there are likely other outcomes/complementary reviews that need to be considered when evaluating impact.

We interviewed a limited number of key informants and aimed to cover a diversity of experiences across sectors, professions and countries. We drew on individuals identified through the literature and a snow-balling approach. Whereas we found a high level of agreement across interviewees’ accounts, both within and between countries, it is possible that a future research agenda could increase the scale and scope of countries and experts covered and that an effort to consider experiences across diverse cancer contexts (types of disease and stages of disease) would offer further learning. For example, despite repeated efforts, the research team was not successful in recruiting a key informant from the USA, at the time of this study. This could have offered important additional insights given the number of tools identified in this study from the USA. We aimed to mitigate this limitation through consultation with representatives at the IBTA World Summit, which included participants from diverse country contexts, including the USA. Evidence from our systematic literature review, selected key informant interviews and wider consultation at a conference of selected attendees, suggest that HRQoL tools *may* improve patient–physician communication, and have the *potential* to improve care, but that they are not widely used in clinical practice. There is a need for further research and stakeholder engagement on how HRQoL tools can achieve impact across different cancer and patient group contexts, in real-world settings. There is also a need for policy, health professional, research and patient communities to strengthen information exchange, support awareness raising, maintain a debate and provide training on tool design, use and interpretation.

## Electronic supplementary material

Below is the link to the electronic supplementary material.

Supplementary material 1 (PDF 170 kb)

Supplementary material 2 (PDF 59 kb)

Supplementary material 3 (PDF 199 kb)

## Footnotes

1 Studies that evaluated a QoL tool to make surgical decisions was beyond the remit of this review; we were primarily interested in general clinical care, and surgery is not always an option for terminal brain cancer patients.

2 Studies that used QoL tools as a predictive tool were not included, as the majority of these types of studies report outcomes that are indirectly applicable to our research question.

3 Given the paucity of literature for the brain patient group, we expanded the search to consider all relevant interventions within all oncology settings, with a view to identifying potentially transferable insights to inform future studies.

4 Five RCTs, two cohorts, two longitudinal studies and one before-and-after study.

5 Five RCTs, four cohort studies, two longitudinal studies and one before-and-after study.

6 There was a debate among the community of academics, healthcare professionals, industry and policy authorities as to what is considered an official QoL assessment tool and what are more informal instruments. The Box highlights the instruments interviewees identified as using in clinical practice.

7 INT02, INT08.

8 11 interviewees: INT01, INT02, INT04, INT05, INT06, INT08, INT09, INT10, INT11, INT13, INT14.

9 INT11.

10 INT06.

11 INT08.

12 INT04.

13 INT03.

14 INT01.

15 INT04, INT07, INT08, INT11, INT14.

16 INT14.

17 INT01, INT03, INT11.

18 For example, changes in waiting areas or in procedures in the clinic, INT11.

19 INT01.

20 INT08, INT10.

21 INT02, INT08, INT09, INT10, INT13.

22 For example, an interviewee reported that patients may report more positively in the presence of a family member/loved one, in an attempt to reassure them.

23 INT08.

24 INT04.

25 INT05, INT06.

26 INT03, INT07, INT11, INT12.

27 INT08, INT10.

28 INT08.

29 INT10.

30 For example, one interviewee saw EQ-5D to be too general to actually measure QoL, while another found it useful for circumstances where patients are unable to fill in a longer questionnaire; the Psychological Screen for Cancer was also described as “not a real QoL measure”.

31 INT04, INT09.

32 INT01, INT02, INT04, INT06, INT07, INT08, INT11, INT12, INT14.

33 INT05, INT15.

34 INT03.
