# Supplementary material for: The use and impact of quality of life assessment tools in clinical care settings for cancer patients, with a particular emphasis on brain cancer: insights from a systematic review and stakeholder consultations
Source: Qual Life Res. 2016 Apr 2;25:2245–56. doi: 10.1007/s11136-016-1278-6 (PMC4980409; doi:10.1007/s11136-016-1278-6)
Supplement: Supplementary file 1 — Supplementary material 1 (PDF 170 kb) [file 11136_2016_1278_MOESM1_ESM.pdf]

Supplementary File 1: Table of QOL Tools

Title: The use and impact of quality of life assessment tools in clinical care settings for cancer patients, with a particular emphasis on brain cancer: insights from a systematic review and stakeholder consultations

AUTHORS

Dr Sarah King[1]
Josephine Exley[2]
Dr Sarah Parks[2]
Dr Sarah Ball[2]
Teresa Bienkowska-Gibbs[2]
Calum MacLure[2]
Emma Harte[2]
Katherine Stewart[2]
Jody Larkin[3]
Dr Andrew Bottomley[4]
Dr Sonja Marjanovic[2]

Corresponding author:
Dr Sonja Marjanovic[2]
smarjano@rand.org
Tel: +44 1223 353 329
Fax: +44(0)1223 358 845

[1] RAND Europe Associate
[2] RAND Europe, Westbrook Centre, Cambridge, CB4 1YG, UK
[3] RAND Corporation, Pittsburgh, USA
[4] European Organisation for Research and Treatment of Cancer (EORTC), Brussels, Belgium

Supplementary File 1: Table of QOL Tools

Instrument	Number of items	Subscales	Administration method	Patient population
**Multi-dimensional QoL questionnaires with patient population specific modules**				
European Organization for Research and Treatment of Cancer (EORTC) – combined use of:				
EORTC QLQ-C30[1] (core questionnaire)	30	5 functional subscales (physical, role, emotional, cognitive, and social functioning)<br>3 symptom scales (fatigue, nausea and vomiting, and pain)<br>1 global health status scale<br>1 overall quality of life scale,<br>6 single items (dyspnoea, insomnia, appetite loss, constipation, diarrhoea, and financial difficulties)	Self-administered	Cancer
EORTC QLQ-BN20[2] (brain-tumour specific module)	20	4 subscales (future uncertainty, visual disorders, motor dysfunction, and communication deficit)<br>7 single items (headache, seizures, drowsiness, hair loss, itchy skin, weakness of legs, and bladder control)	Self-administered	Brain cancer
Functional Assessment of Cancer Therapy-Brain (FACT-Br) – comprising:				
FACT-General (FACT-G) Version 4[3] (core questionnaire)	27	4 subscales (physical, social/family, emotional, and functional well-being)	Self-administered	Cancer

[1] Aaronson NK, Ahmedzai S, Bergman B, et al. The European Organization for Research and Treatment of Cancer QLQ-C30: a quality-of-life instrument for use in international clinical trials in oncology. J Natl Cancer Inst. 1993;85:365–376.
[2] Osoba D, Aaronson NK, Muller M, et al. The development and psychometric validation of a brain cancer quality-of-life questionnaire for use in combination with general cancer-specific questionnaires. Qual Life Res. 1996;5:139–150.
[3] Cella DF, Tulsky DS, Gray G, et al. The Functional Assessment of Cancer Therapy scale: development and validation of the general measure. J Clin Oncol. 1993;11:570–579.

FACT-brain subscale[4] (brain-cancer specific module)	23	1 subscale (brain cancer related concerns including concentration, memory, seizures, eyesight, hearing, speech, personality, expression of thoughts, weakness, coordination, and headaches)	Self-administered	Brain cancer
MD Anderson Symptom Inventory (MDASI)[5] combined use of:				
MD Anderson Symptom Inventory (MDASI) (core questionnaire)	19	2 subscales (severity of symptoms, interference of symptoms with activities of daily living)	Self-administered, interview or via telephone-based interactive voice response (IVR) system	Cancer
MDASI-BT[6] (brain-tumour specific module)	9	1 subscale (brain cancer related symptoms - weakness, difficulty understanding, difficulty speaking, seizures, difficulty concentrating, vision, change in appearance, change in bowel pattern, and irritability)	Self-administered, interview or via telephone-based interactive voice response (IVR) system	Brain cancer
**Other multi-dimensional QoL questionnaires**				
Rotterdam Symptom Check List (RSCL)[7]	39	4 subscales (physical symptom distress, psychological distress, activity level, overall global life quality)	Self-administered	Cancer
Functional Living Index-Cancer (FLIC)[8]	22	5 domains (physical well-being and ability, emotional state, sociability, family situation, nausea)	Self-administered	Cancer
Short-Form 36 questionnaire[9]	36	8 subscales (vitality, physical functioning, bodily pain, general health perceptions, physical role functioning, emotional role functioning, social role functioning, mental	Self-administered	Generic

[4] Weitzner MA, Meyers CA, Gelke CK, et al. The Functional Assessment of Cancer Therapy (FACT) scale. Development of a brain subscale and revalidation of the general version (FACT-G) in patients with primary brain tumors. Cancer. 1995;75:1151–1161.

[5] Cleeland CS, Mendoza TR, Wang XS. Assessing symptom distress in cancer patients: the M.D. Anderson Symptom Inventory. Cancer. 2000;89:1634–1646.

[6] Armstrong TS, Mendoza T, Gning I, et al. Validation of the M.D. Anderson Symptom Inventory Brain Tumor Module (MDASI-BT) J Neurooncol. 2006;80:27–35.

[7] de Haes JC, van Knippenberg FC and Neijt JP: Measuring psychological and physical distress in cancer patients: structure and application of the Rotterdam Symptom Checklist. Br J Cancer *62*: 1034-1038, 1990.

[8] Schipper H, Clinch J, McMurray A and Levitt M: Measuring the quality of life of cancer patients: the Functional Living Index- Cancer: development and validation. J Clin Oncol *2*: 472-483, 1984.

[9] McHorney CA, Ware JE Jr and Raczek AE: The MOS 36-Item Short-Form Health Survey (SF-36): II. Psychometric and clinical tests of validity in measuring physical and mental health

		health)		
Nottingham Health Profile[10]	45	6 subscales (energy level , pain, emotional reaction, sleep, social isolation, physical abilities) and 7 items on life areas affected (work, looking after the home, social life, home life, sex life, interests and hobbies, vacations)	Self-administered	Generic
**Single item or brief multi-item indices of QoL**				
Linear Analog Scale Assessment (LASA)[11 12]	5	5 domains (1 item per domain - physical wellbeing, emotional well-being, spiritual well-being, intellectual well-being and overall QOL) - patients directly score their quality of life or functioning on a 100mm horizontal line	Self-administered	Brain-cancer
Spitzer Quality of Life Index (QLI)[13]	5	5 domains (health, activity, daily living, outlook, support)	Physician-administered	Cancer
The schedule of evaluation of individual quality of life–direct weighting (SEIQoL-DW)[14]	5	Covers 5 areas of life selected by the patient to be important to QoL	Interview-based	Generic
EuroQol 5D	6	5 subscales (mobility, self-care, usual activities, pain/discomfort and anxiety/depression) and self-rated health on a visual analogue scale	Self-administered	Generic
Sintonen's 15D				
**Instruments measuring a single factor of QoL***				

constructs. Med Care *31*: 247-263, 1993.

[10] Hunt SM, McKenna SP, McEwen J, Williams J and Papp E: The Nottingham Health Profile: subjective health status and medical consultations. Soc Sci Med *15*: 221-229, 1981.

[11] Locke DE, Decker PA, Sloan JA, et al. Validation of single-item linear analog scale assessment of quality of life in neuro-oncology patients. J Pain Symptom Manage. 2007;34:628–638.

[12] Sloan JA, Aaronson N, Cappelleri JC, Fairclough DL, Varricchio C. Assessing the clinical significance of single items relative to summated scores. Mayo Clin Proc. 2002;77:479–487.

[13] Spitzer WO, Dobson AJ, Hall J, Chesterman E, Levi J, Shepherd R, Battista RN and Catchlove BR: Measuring the quality of life of cancer patients: a concise QL-index for use by physicians. J Chronic Dis *34*: 585-597, 1981.

[14] Hickey AM, Bury G, O'Boyle CA, Bradley F, O'Kelly FD, Shannon W (1996) A new short form individual quality of life measure (SEIQoL-DW): Application in a cohort of individuals with HIV/AIDS. Br Med J (Clin Res Ed) 313:29–33

The Karnofsky performance status scale[15]	1	100-point rating index to assess patients' physical and functional performance abilities (range from 0 (dead) to 100 (no impairment, normal activity)).	Physician-administered	Cancer
Distress thermometer[16]	1	Single item visual analogue scale to screen for psychological distress	Self-administered	Cancer
Symptom Distress Scale (SDS)[17]	13	Physical concerns in cancer patients	Self-administered	Cancer
Profile of Mood States-Short Form (POMS-SF)[18]	30	6 subscales (tension/anxiety, vigor/activity, fatigue/inertia, depression/dejection, confusion/bewilderment, and anger/hostility)	Self-administered	Cancer
The hospital anxiety and depression scale[19]	14	Two 7-item scales (anxiety, depression)	Self-administered	Generic
Mini-Mental State Examination [20]	30	Screening test for dementia and cognitive impairment	Physician-administered	Generic
Multidimensional Fatigue Inventory				
The McGill Pain Questionnaire				
Activities of Daily Living				

*These instruments are not strictly measuring QoL – so debatable as to whether we would include.

[15] Karnofsky DA, Abelman WH, Craver LF, Burchenal JH. The use of nitrogen mustards in the palliative treatment of carcinoma. Cancer. 1948;1:634–656.
[16] Keir, S. T., et al. (2008). "Screening for distress in patients with brain cancer using the NCCN's rapid screening measure." Psychooncology **17**(6): 621-625.
[17] McCorkle R, Benoliel J. Symptom distress, current concerns, and mood disturbance after diagnosis of life-threatening disease. Soc Sci Med 1983;17:431–438.
[18] Baker F, Denniston M, Zabora J, et al. A POMS short form for cancer patients: psychometric and structural evaluation. Psychooncology 2002;11:273–281.
[19] Zigmond AS and Snaith RP: The hospital anxiety and depression scale. Acta Psychiatr Scand 67: 361-370, 1983.
[20] Tombaugh TN, McIntyre NJ. The Mini-Mental State Examination: a comprehensive review. J Am Geriatr Soc. 1992;40:922–935.
