# Supplementary material for: The use and impact of quality of life assessment tools in clinical care settings for cancer patients, with a particular emphasis on brain cancer: insights from a systematic review and stakeholder consultations
Source: Qual Life Res. 2016 Apr 2;25:2245–56. doi: 10.1007/s11136-016-1278-6 (PMC4980409; doi:10.1007/s11136-016-1278-6)
Supplement: Supplementary file 2 — Supplementary material 2 (PDF 59 kb) [file 11136_2016_1278_MOESM2_ESM.pdf]

Supplementary File 2: Search Terms

Title: The use and impact of quality of life assessment tools in clinical care settings for cancer patients, with a particular emphasis on brain cancer: insights from a systematic review and stakeholder consultations

AUTHORS

Dr Sarah King[1]
Josephine Exley[2]
Dr Sarah Parks[2]
Dr Sarah Ball[2]
Teresa Bienkowska-Gibbs[2]
Calum MacLure[2]
Emma Harte[2]
Katherine Stewart[2]
Jody Larkin[3]
Dr Andrew Bottomley[4]
Dr Sonja Marjanovic[2]

Corresponding author:
Dr Sonja Marjanovic[2]
smarjano@rand.org
Tel: +44 1223 353 329
Fax: +44(0)1223 358 845

[1] RAND Europe Associate
[2] RAND Europe, Westbrook Centre, Cambridge, CB4 1YG, UK
[3] RAND Corporation, Pittsburgh, USA
[4] European Organisation for Research and Treatment of Cancer (EORTC), Brussels, Belgium

Search terms

PubMed

((Cancer[title/abstract] OR neoplasms[MeSH] OR glioblastoma)
AND
("quality of life"[title/abstract] OR "health related quality of life"[title/abstract] OR QOL[title/abstract] OR HRQOL[title/abstract])
AND
(questionnaire*[title/abstract] OR survey*[title/abstract] OR measures[title/abstract]))

OR

((EORTC[title/abstract] AND QLQ-C30[title/abstract])
OR

(EORTC[title/abstract] AND BN-20 [title/abstract)
OR
((("Symptom Inventory"[title/abstract] AND ("Brain Tumor"[title/abstract] OR “Brain Tumour” [title/abstract])) AND “MD Anderson”[title/abstract])))
OR
“MDASI-BT”
OR
((("functional assessment"[Title/Abstract]) AND "cancer therapy"[Title/Abstract]))))

*removed animal

Embase
(pub types: article and “article in press” only)

(cancer*:ab,ti OR neoplasm*:ab,ti OR glioblastoma*:ab,ti)
AND
(“quality of life”:ab,ti OR “health related quality of life”:ab,ti OR QOL:ab,ti OR HRQOL:ab,ti)
AND
(questionnaire*:ab,ti OR survey*:ab,ti OR measures:ab,ti)

OR

“EORTC QLQ-C30”:ab,ti OR “European Organization for Research and Treatment of Cancer Quality of Life Questionnaire-Core 30”:ab,ti OR “EORTC BN-20”:ab,ti OR “European Organization for Research and Treatment of Cancer Quality of Life Questionnaire BN-20”:ab,ti OR “Functional Assessment of Cancer Therapy-Brain Subscale”:ab,ti OR “FACT-Brs”:ab,ti OR “Functional Assessment of Cancer Therapy-General”:ab,ti OR “FACT-G”:ab,ti OR “MD Anderson Symptom Inventory-Brain Tumour”:ab,ti OR “MDASI-BT”:ab,ti OR “MD Anderson Symptom Inventory-Brain Tumor”:ab,ti

NOT (Cats:ti OR canine:ab,ti OR dogs:ab,ti OR dog:ab,ti OR mice:ab,ti OR mouse:ab,ti)

Web of Science
(Science Citation Index Only)

TS= (cancer* OR neoplasm* OR glioblastoma*)
AND
TS= (“quality of life” OR “health related quality of life” OR QOL OR HRQOL)
AND
TS=(questionnaire* OR survey* OR measures)

OR

TS= (“EORTC QLQ-C30” OR “European Organization for Research and Treatment of Cancer Quality of Life Questionnaire-Core 30” OR “EORTC BN-20” OR “European Organization for Research and Treatment of Cancer Quality of Life Questionnaire BN-20” OR “Functional Assessment of Cancer Therapy-Brain Subscale” OR “FACT-Brs” OR “Functional Assessment of Cancer Therapy-General” OR “FACT-G” OR “MD Anderson Symptom Inventory-Brain Tumour” OR “MD Anderson Symptom Inventory-Brain Tumor” OR “MDASI-BT”)

NOT

(TI=(mice OR mouse OR dog OR dogs OR canine OR cat OR cats OR rodent OR rodents OR rat OR rats))

Cochrane
((cancer* or neoplasm* or glioblastoma*):ab or (cancer* or neoplasm* or glioblastoma*):ti (Word variations have been searched)
AND
("quality of life" or "health related quality of life" or QOL or HRQOL):ab or ("quality of life" or "health related quality of life" or QOL or HRQOL):ti (Word variations have been searched)
AND
(questionnaire* or survey* or measures):ab or (questionnaire* or survey* or measures):ti in Trials (Word variations have been searched))

OR

("EORTC QLQ-C30" or "European Organization for Research and Treatment of Cancer Quality of Life Questionnaire-Core 30":ab or "EORTC QLQ-C30" or "European Organization for Research and Treatment of Cancer Quality of Life Questionnaire-Core 30":ti
OR
"Functional Assessment of Cancer Therapy-Brain Subscale" or "FACT-Brs" or "Functional Assessment of Cancer Therapy-General" or "FACT-G" or "MD Anderson Symptom Inventory-Brain Tumour" or "MD Anderson Symptom Inventory-Brain Tumor" or "MDASI-BT":ti or "Functional Assessment of Cancer Therapy-Brain Subscale" or "FACT-Brs" or "Functional Assessment of Cancer Therapy-General" or "FACT-G" or "MD Anderson Symptom Inventory-Brain Tumour" or "MD Anderson Symptom Inventory-Brain Tumor" or "MDASI-BT":ab
OR
"EORTC QLQ-C30" or "European Organization for Research and Treatment of Cancer Quality of Life Questionnaire-Core 30":ab or "EORTC QLQ-C30" or "European Organization for Research and Treatment of Cancer Quality of Life Questionnaire-Core 30":ti
OR
EORTC BN-20" or "European Organization for Research and Treatment of Cancer Quality of Life Questionnaire BN-20":ti or "EORTC BN-20" or "European Organization for Research and Treatment of Cancer Quality of Life Questionnaire BN-20":ab
