# Supplementary material for: The use and impact of quality of life assessment tools in clinical care settings for cancer patients, with a particular emphasis on brain cancer: insights from a systematic review and stakeholder consultations
Source: Qual Life Res. 2016 Apr 2;25:2245–56. doi: 10.1007/s11136-016-1278-6 (PMC4980409; doi:10.1007/s11136-016-1278-6)
Supplement: Supplementary file 3 — Supplementary material 3 (PDF 199 kb) [file 11136_2016_1278_MOESM3_ESM.pdf]

Supplementary File 3: Characteristics and results of the included studies

Title: The use and impact of quality of life assessment tools in clinical care settings for cancer patients, with a particular emphasis on brain cancer: insights from a systematic review and stakeholder consultations

AUTHORS

Dr Sarah King[1]
Josephine Exley[2]
Dr Sarah Parks[2]
Dr Sarah Ball[2]
Teresa Bienkowska-Gibbs[2]
Calum MacLure[2]
Emma Harte[2]
Katherine Stewart[2]
Jody Larkin[3]
Dr Andrew Bottomley[4]
Dr Sonja Marjanovic[2]

Corresponding author:
Dr Sonja Marjanovic[2]
smarjano@rand.org
Tel: +44 1223 353 329
Fax: +44(0)1223 358 845

[1] RAND Europe Associate
[2] RAND Europe, Westbrook Centre, Cambridge, CB4 1YG, UK
[3] RAND Corporation, Pittsburgh, USA
[4] European Organisation for Research and Treatment of Cancer (EORTC), Brussels, Belgium

Supplementary File 4: Characteristics and results of the included studies

Reference<br><br>Study country<br><br>Risk of bias	Patient group	Study type<br><br>Setting<br><br>Final assessment	Intervention and control group (sample sizes)	Results (as reported by the study authors)			
				**Physician-patient communication**	**Patient management**	**Patients' self-reported HRQL**	**Patient satisfaction with care/treatment**
Álvarez-Maestro M, et al. 2014<br><br>Spain<br><br>High risk of bias	Men with metastatic, symptomatic prostate cancer	Prospective longitudinal study<br><br>Clinics and hospitals<br><br>Outcome assessed at 6 months	Patients filled out a PROSQoLI questionnaire at each visit (n=709 patients and 126 physicians)	The proportion of physicians rating the usefulness of the tool as high for this outcome was 73.4% at baseline compared to 79.1% at follow-up (p<0.01). The tool was most useful for patients with no education (OR 2.89 [95%CI 1.10, 7.59]) or low education (OR 2.29 [95% CI 1.08, 4.86]).	The proportion of physicians rating the usefulness of the tool as high for making clinical decisions increased from 66.1% at baseline to 70.7% at follow-up (p<0.01). Such clinical decisions included establishing the most appropriate treatment option, deciding on possible changes in dose or guidelines, establishing the frequency of the follow-up visits, and referral for other health problems (e.g. to psychiatry).	NA	NA
Berry DL, et al. 2011<br><br>USA<br><br>Unclear risk of bias	Men and women with various cancer diagnoses (breast, GI, genitourinary, gynaecologic, head and neck, leukaemia, lung, lymphoma, myeloma)	RCT<br><br>Ambulatory clinics<br><br>Outcome assessed at 4 and 6 weeks	Patients filled out (ESRA-C) – a summary of which was provided to the clinical team (n=327 [295 analysed]) vs. no summary (n=333 [295 analysed])	There were no significant differences in the proportion of patients in each group who discussed 23 symptom and QoL issues (SQLIs) with their physician, with the exception of emotional function which was higher in the control group (8.4% vs 4.1%, P=0.03). Modelling demonstrated that the likelihood of the SQLIs being discussed depended on whether	NA	NA	NA

Reference<br><br>Study country<br><br>Risk of bias	Patient group	Study type<br><br>Setting<br><br>Final assessment	Intervention and control group (sample sizes)	Results (as reported by the study authors)			
				**Physician-patient communication**	**Patient management**	**Patients' self-reported HRQL**	**Patient satisfaction with care/treatment**
				SQUI was first reported as problematic.			
Detmar SB, et al. 2002a<br><br>The Netherlands<br><br>Unclear risk of bias	Men and women undergoing palliative chemotherapy (breast, colorectal, gynaecological, lymphoma, other)	RCT (cross-over); In this study, the physicians were randomised (n=10), not the patients<br><br>Outpatient chemotherapy clinic<br><br>Outcome assessed at '4th visit'	Patients completed the EORTC QLQ-30 in the waiting room immediately before a visit with their physician before the start of each cycle of chemotherapy (n=145 patients [114 analysed]; 5 physicians) vs. no questionnaire (n=128 patients [100 analysed]; 5 physicians)	Composite communication score: intervention group mean 4.5 (SD 2.3); control group mean 3.7 (SD 1.9); effect size 0.38 (95% CI: 0.10 to 0.66), P=0.01 (n=104 in intervention group and n=95 in the control group).<br><br>Of 12 HRQoL issues, social functioning (22% vs 11%, p=0.05), fatigue (54% vs 37%, p=0.02) and dyspnoea (23% vs 13%, p=0.02) were discussed with more patients who completed the EORTC QLQ-30 questionnaire compared to those who did not.	Mean number of HRQL-related patient management actions taken per patient: Intervention group mean 0.6 (SD not reported); Control group mean 0.5 (SD not reported). No statistically significant differences between groups were observed in the prescription of medications, ordering of tests, or referrals to other health care practitioners (data not reported).<br><br>A greater percentage of patients who received the intervention received counselling from their physician on how to manage their health problems compared with those in the control group (23% vs. 16%, P=0.05) (n=104 in intervention group and n=95 in the control group).	There were no statistically significant between-group differences for any of the SF-36 scales (physical functioning, social functioning, role physical, role emotional, bodily pain, vitality, and mental health).	5-item Patient Satisfaction Questionnaire C: Overall mean scores were high in both groups (data not reported).<br><br>For individual items, there was a statistically significant difference between groups on the degree of emotional support received: Intervention group: mean 4.3 (SD 0.7); Control group: mean 4.0 (SD 0.9); P=0.05.
Engelen V, et al. 2012<br><br>The Netherlands<br><br>High risk of bias	Children (0-18 years of age) with any type of cancer (leukaemia, lymphoma, brain tumour, solid tumour, bone tumour, or other) 0-3 months after treatment	Sequential cohort design<br><br>Medical centre<br><br>Outcome assessed 'after 3	Patients' HRQOL scores (Quality of Life in Childhood Oncology [QLIC-ON] PROfile) were given to the oncologist. Training sessions on the tool were provided (n=94) vs. no	Communication about HRQOL domains (score from 0 [not discussed] to 100 [extensively discussed]: Emotional and psychosocial functioning were more extensively discussed in	There were no differences in psychosocial referrals between the intervention and control groups: consultation 1 intervention 3.2% vs. 2.2% control, p=0.44; consultation 2 intervention 2.3% vs. 4.7% control, p=0.26; consultation 3	(Parent or child assessed) The authors stated that "children aged 5-7 participating in the intervention group, scored significantly better at follow-up than controls	There was no difference between the intervention and control groups (regression coefficients for intervention vs. control for parents satisfaction with their child's

Reference<br><br>Study country<br><br>Risk of bias	Patient group	Study type<br><br>Setting<br><br>Final assessment	Intervention and control group (sample sizes)	Results (as reported by the study authors)			
				Physician-patient communication	Patient management	Patients' self-reported HRQL	Patient satisfaction with care/treatment
	ended, or 6 months after treatment ended for children with stem cell transplantations	consultations'	questionnaire (n=99)	the intervention group than in the control group (intervention mean score 47 vs. control 33; 64 vs. 57, respectively, P < 0.05 for both); there were no differences in scores for physical, social, or cognitive domains.	intervention 2.0% vs. 2.6% control, p=0.85.	on the subscales of self-esteem (B = 10.17; P<0.05), family activities (B = 11.11; P<0.05), and psycho-social summary (B = 6.45; P<0.01). In 0-4 and 8-18 year old children and adolescents, no differences between the intervention and control groups were observed."	oncologist was B = 1.15, p = 0.52).
Hilarius DL, et al. 2008<br><br>The Netherlands<br><br>High risk of bias	Patients who had received one cycle of chemotherapy (breast, lung, colorectal, urogenital, gynaecologic, Non Hodgkin lymphoma, Hodgkin disease, sarcoma, other)	Sequential cohort design<br><br>Outpatient chemotherapy clinic<br><br>Outcome assessed at '4th visit'	Patients completed the EORTC QLQ-C30 (version 3.0) via touch screen computer and, if applicable, a condition-specific EORTC module after each visit (n=148 [111 analysed] vs. usual care (n=150 [108 analysed])	Nurse-patient communication: Composite communication score: intervention mean 4.8 (SD 3.3); control group mean 3.8 (SD 2.3), P=0.009.	Percentage of cases with HRQL-related medication prescription (intervention vs. control = 76% vs. 84%, p=0.11), test ordering (30% vs. 40%, p=0.43), referrals to other healthcare providers (50% vs. 48%, p=0.94), modification/cessation of chemotherapy (32% vs 29%, p=0.55), or HRQL related advice/counselling (74% vs. 62%, p=0.06).<br><br>The mean number of HRQL-related medical record notations was 24 in the intervention group and 20 in the control group (p<0.01).	No statistically significant between-group differences at the fourth visit on any of the SF-36 scales or on the FACT breast or colon cancer subscales (data were not reported).	No statistically significant group differences were observed for any of the patient satisfaction scales (based in part on Patient Satisfaction Questionnaire), with patients in both the intervention and control groups reporting high levels of satisfaction (data not reported).

Reference<br><br>Study country<br><br>Risk of bias	Patient group	Study type<br><br>Setting<br><br>Final assessment	Intervention and control group (sample sizes)	Results (as reported by the study authors)			
				Physician-patient communication	Patient management	Patients' self-reported HRQL	Patient satisfaction with care/treatment
Klinkhammer-Schalke, M, et al. 2012<br><br>Bavaria, Germany<br><br>Low risk of bias	Female breast cancer patients (newly diagnosed)	RCT<br><br>Hospitals<br><br>Outcome assessed at 6 months	Novel care pathway including a diagnosis of 'diseased' QoL (a rating of less than 50 out of 100 - based on a profile derived from EORTC QLQ-C30 and QLQ-BR23 questionnaires, and an expert report) (n=100) vs. standard care (n=100)	NA	At 3 months, at least one treatment for QOL had been ordered for 42% of patients in the intervention group (n=92) compared with 35% in the control group (n=99). At 6 months, these figures were 35% vs. 39% (no significant differences for both time points). Rates of coping strategies and counselling given were: 21% vs 12%, p<0.06 at 3 months, and 19% vs. 10% at 6 months; rates for psychotherapy were 10% vs. 1%, p<0.05 at 3 months and 3% vs. 3% at 6 months; rates for physiotherapy were 18% vs. 25% at 3 months, and 16% vs. 30%, p<0.02 at 6 months.	After six months, 47/84 (56%) of patients in the intervention group and 60/85 (71%) of patients in the control group showed diseased QoL in at least one dimension of the QoL profile (p=0.048), corresponding to 21% relative risk reduction (95% CI: 0 to 37).	NA
McLachlan, SA, et al. 2001<br><br>Australia<br><br>High risk of bias	Patients with lung, head and neck, genitourinary, skin, or other cancers of any clinical stage	RCT<br><br>Medical oncology clinic<br><br>Outcome assessed at 2 and 6 months	Patients completed self-reported questionnaires (Cancer Needs Questionnaire-short form [CNQ], EORTC QLQ-C30, and Beck Depression Inventory (BDI) Short form) via a touch-screen computer, the results of which were made available	NA	NA	There was no difference between the two arms for any of the EORTC QLQ-C30 subscales at 6 months: Cognitive functioning (Mean difference in change from baseline scores (MD) 1.4 [95% CI: -2.6 to 5.5]); Emotional functioning	No significant differences between the two arms with respect to levels of satisfaction (data not reported). In both groups, treatment satisfaction was generally very high.

Reference<br><br>Study country<br><br>Risk of bias	Patient group	Study type<br><br>Setting<br><br>Final assessment	Intervention and control group (sample sizes)	Results (as reported by the study authors)			
				Physician-patient communication	Patient management	Patients' self-reported HRQL	Patient satisfaction with care/treatment
			immediately to the healthcare team to formulate an individualised management plan (n=296 [213 analysed]) vs. standard care (n=154 [106 analysed])			(MD 1.4 [95% CI: -2.9 to 5.7]); Global health status/QOL (MD 2.5 [95% CI: -2.2 to 7.1]); Physical functioning (MD 4.6 [95% CI: -0.1 to 9.4]); Role functioning (MD 2.0 [95% CI: -5.6 to 9.5]); Social functioning (MD 0.9 [95% CI: -4.2 to 5.9]).	
Mills ME, et al. 2009<br><br>UK (Northern Ireland)<br><br>High risk of bias	Men and women with inoperable lung cancer	RCT<br><br>Hospital<br><br>Outcome assessed at 2 and 4 months	Patients filled out a structured diary (EORTC QLQ-C30 and its associated module for lung cancer [Quality of Life Questionnaire LC13]) for 16 weeks (n=57 [n=28 analysed for primary outcome - TOI]) vs. no diary - standard care (n=58 [25 analysed for the primary outcome)	There were no significant differences in the proportion of patients in each group (n=23 and n=27) who discussed social relationships (87% vs 85%), financial issues (96% vs 100%), work problems (96% vs 100%), or family problems (91% vs 96%).	NA	There was no significant difference in QOL change scores (using the Trial Outcome Index [the primary outcome]) between the diary and non-diary groups (mean difference in change in score from 0-4 months = -5.5, 95% CI -12.8 to 1.9, p=0.14). A borderline significant difference was observed for the total FACT-L score (mean difference in change in score from 0-4 months = -9.7, 95% CI -19.4 to 0.0, p=0.05), but no significant differences	There were no significant differences in the proportion of patients in each group (n=25 and n=23) who were satisfied with their care: care met patients' needs (88% vs 96%); patients' expectations (88% vs 96%); patient/family involved in decision making (72% vs 78%); satisfaction with information given (84% vs 87%); satisfaction with emotional support (84% vs. 78%); 'good' quality of care (84% vs 91%).

Reference<br><br>Study country<br><br>Risk of bias	Patient group	Study type<br><br>Setting<br><br>Final assessment	Intervention and control group (sample sizes)	Results (as reported by the study authors)			
				Physician-patient communication	Patient management	Patients' self-reported HRQL	Patient satisfaction with care/treatment
						between groups were observed for FACT-G, LCS, or PQLI overall score.	
Nicklasson M, et al. 2013<br><br>Sweden<br><br>Low risk of bias	Men and women with lung cancer or mesothelioma not amenable to curative treatment	RCT<br><br>Outpatient department of a hospital<br><br>Outcome assessed after 2 to 3 months	Patients answered a computerized version the EORTC QLQ-C30, version 3 and the complementary lung cancer module LC13), using a digital table interface – and a summary report was presented to the physician prior to consultation (n=85) vs. no information presented to the physician (n=88)	Issues regarding emotional functioning (mean 3.9 'statements' (SD 4.3) vs. 2.4 (SD 2.3), p= 0.015) and a function sum (mean 9.2 (SD 5.9) vs. 6.9 (SD 4.3), p= 0.01) were more frequently discussed in the intervention group (N=80) compared with the control group (N=85). There were no significant differences between groups for 13 other issues.	The number of diagnostic and therapeutic interventions directed to emotional and social concerns was higher in the intervention group (N=84) compared with the control group (N=87) (mean, 0.43 (SD 0.80) vs. 0.15 (SD 0.36) interventions per patient for emotional concerns; p=0.0036 and mean, 1.17 (SD 1.32) vs. 0.74 (0.88) interventions per patient for social concerns; p=0.013). No significant differences were observed in the number of diagnostic actions taken for the following: pain, fatigue, anorexia, nausea, constipation, cough, neurological symptoms, sleep difficulties, other symptoms.	NA	NA
Rosenbloom SK, et al. 2007<br><br>USA<br><br>High risk of bias	Men and women with advanced metastatic breast, lung or colorectal cancer, who were receiving chemotherapy.	RCT<br><br>Medical centre<br><br>Outcome assessed at 3 and 6 months	Patients completed HRQL questionnaire (FACT-G), and were interviewed by a research nurse - which was then relayed to the treating nurses (n=69	NA	There were no significant differences between the three groups in the mean composite clinical treatment change (range from 0 to 5, n=200): interview group vs. questionnaire only group vs. usual care: 0.97 (SD	There were no significant differences between the three groups (as measured by the FLIC total score at 6 months): interview group: mean 115.8 (SD	There were no significant differences between the three groups (as measured by PSQ-III General Satisfaction and Satisfaction with

Reference<br><br>Study country<br><br>Risk of bias	Patient group	Study type<br><br>Setting<br><br>Final assessment	Intervention and control group (sample sizes)	Results (as reported by the study authors)			
				**Physician-patient communication**	**Patient management**	**Patients' self-reported HRQL**	**Patient satisfaction with care/treatment**
			[51 analysed]) vs. questionnaire and no interview (n=73 [51 analysed]) vs. usual care (n=71 [52 analysed]).		1.3) vs. 1.2 (SD 1.2) vs. 0.79 (SD 1.0), p=0.17.	22.9); questionnaire only group: 113.3 (SD 24.5); usual care: 112.2 (SD 21.4).	Communication subscales at 6 months (data presented in a figure only).
Snyder CF, et al. 2013<br><br>USA<br><br>High risk of bias	Patients (>21 years old) with breast or prostate cancer (at any stage)	Prospective longitudinal study<br><br>Ambulatory practices<br><br>Outcome assessed 'after 3 visits'	Patients were assigned to complete the PRO questionnaires online every 2 weeks (n=52 [n=47 analysed])	The most frequently discussed issues by the physicians (of 18 dominant issues identified by the patients) were systematic therapy (discussed with 89% of patients with this issue) and fatigue (80%); the least discussed was sexual function (6%).	Of the 18 dominant issues, 20% of patients who discussed a physical function issue with their physician were prescribed medication; 25% of patients who discussed pain were prescribed medication and 38% were referred; 25% who discussed fatigue were prescribed medication; 29% who discussed body image were referred; 12% who discussed systemic therapy were prescribed medication and 19% were referred. The authors stated that the most common actions taken in response to identified issues were providing information and/or advice.	NA	Patient feedback: 39% reported that care quality improved.
Taenzer P, et al. 2000<br><br>Canada<br><br>High risk of bias	Men and women with a diagnosis of primary, secondary or metastatic lung cancer of any stage.	Cohort study<br><br>Outpatient clinic<br><br>Follow-up post-appointment	Patients completed a computerised version of the EORTC QLQ-C30 prior to appointment, and information was provided to clinic staff (n=27) vs. usual care (n=26)	Based on interview data from patients, there was no significant difference between groups in the mean number of EORTC items discussed - which were endorsed on the patient questionnaire: Intervention 13.1 (SD	Based on interview data from patients, there was a significant difference in favour of the intervention group regarding the mean number of EORTC items addressed during the clinic appointment: Intervention 6.4 (SD 4.1) vs Control 2.5 (SD 2.9), p<0.01.	NA	No significant difference between groups on any of the 11 items or 3 sub-scales of the Patient Satisfaction Questionnaire.

Reference<br><br>Study country<br><br>Risk of bias	Patient group	Study type<br><br>Setting<br><br>Final assessment	Intervention and control group (sample sizes)	Results (as reported by the study authors)			
				**Physician-patient communication**	**Patient management**	**Patients' self-reported HRQL**	**Patient satisfaction with care/treatment**
				5.5) vs control 10.6 (SD 6.0).<br><br>A medical record audit, however, revealed that a greater mean number of EORTC categories were charted in the intervention group patients compared with control group patients: 1.1 (SD 0.9) vs. 0.7 (SD 0.9), p<0.1.	A medical record audit, however, revealed that there was no significant differences in the number of actions taken between the intervention and control, 0.8 (SD 0.8) vs. 0.5 (SD 0.7).		
Velikova G., et al. 2002<br><br>UK<br><br>High risk of bias	Male and female adult oncology patients receiving chemotherapy or biological therapy (ovarian or malignant melanoma)	Before and after<br><br>Outpatient clinic<br><br>Follow-up post-appointment	Computer-administered EORTC QLQ-C30 and the Hospital Anxiety and Depression Scale (HADS) with immediate feedback to clinicians (n=34, [n=28 analysed])	Two out of seven topics were discussed in more consultations after the intervention - daily activities: 13 before vs. 23 after, p=0.007, and emotional problems: 16 vs. 24, p=0.035.	54% physicians said the tool contributed to some management decisions; 1 changing chemotherapy, 2 readjusting drugs, 1 blood transfusion, 3 counselling about lifestyle, 1 reassurance, 1 discussion of depression.	NA	No difference between consultations - patient satisfaction was high at baseline (median and range for baseline and intervention consultations were 82.5 (74-85) and 83.5 (70-85 respectively) (the range of possible total scores on the instrument ranges from 17 to 85).
Velikova G., et al. 2004<br><br>UK<br><br>High risk of bias	Male and female adult oncology patients (breast, gynaecologic , renal, bladder, sarcoma, melanoma, other)	RCT<br><br>Oncology clinic<br><br>Outcome assessed at 6 months	Completion of touch-screen EORTC QLQ-C30 questionnaire and the Hospital Anxiety and Depression Scale (HADS) with feedback of results to physicians (n=144) vs. completion	The number of EORTC QLQ-C30 symptoms discussed (out of a possible 7) was higher in the intervention group (n=103) compared with the control group (n=56) (mean 3.3 (SD 1.63) vs.	No effect on patient management was detected: mean number of medical decisions: intervention 1.9 (SD 0.89) vs. attention control group 2.0 (SD 1.02) vs. control 2.1 (SD 0.98).	FACT-G scores were significantly better in the intervention arm (n=84) compared to the control arm (n=45) (estimate of effects 8.01 (SE 2.84), p=0.006), but were not	Between 79% - 89% of patients rated care as very good or excellent regardless of study arm, and between 89%-95% of patients felt the doctor met their

Reference<br><br>Study country<br><br>Risk of bias	Patient group	Study type<br><br>Setting<br><br>Final assessment	Intervention and control group (sample sizes)	Results (as reported by the study authors)			
				Physician-patient communication	Patient management	Patients' self-reported HRQL	Patient satisfaction with care/treatment
			of HRQOL scores, but no feedback to physicians (n=70) vs. usual care (n=72)	2.7 (SD 1.53), p=0.03).<br><br>There was no significant difference between the groups in the number of functions (physical, emotional, cognitive, social role) discussed between the groups (intervention vs. vs. control): 1.57 vs. 1.36, p=0.084).		significantly different between the intervention and attention-control arm (n=35) (estimate of effects 0.76 (SE 3.07), p=0.80).	expectations.
Wolfe J., et al. 2014<br><br>USA<br><br>Low risk of bias	Children aged 2 years or older and teens with advanced cancer (haematologic malignancy, brain tumour, solid tumour)	RCT<br><br>Cancer care centres<br><br>Outcome assessed at 6 months	Computer-based HRQOL data collection (PediQUEST) which generated feedback reports and email alerts to oncologists and families (n=51 [49 analysed]) vs. no feedback (n=53 [49 analysed])	NA	The reports contributed at least sometimes to a providers' decision to initiate a psychosocial (56%), pain (34%), social work (33%), or palliative care (29%), consult and to discuss goals with families (36%). Few agreed that reports changed their views about patient's treatment goals (results were not presented by group for this outcome).	Feedback did not significantly affect the average PQ-MSAS total score (mean score difference -0.7 [95% CI: -2.9 to 1.6], p=0.57), PedsQL4.0 total score (mean score difference 1.5 [95% CI: -4.2 to 7.2], p=0.61); or Sickness score (mean score difference -1.7 [95% CI: -7.9 to 4.5], p=0.59) during 20 weeks of follow-up.	NA
